# Two-Step *Vibrio parahaemolyticus* Challenge Reveals Transcriptional Reprogramming of Trained Immunity in Shrimp Hemocytes

**DOI:** 10.3390/biology15120956

**Published:** 2026-06-18

**Authors:** Zhongying Li, Shihao Li, Xinjia Lv, Fuhua Li

**Affiliations:** 1 School of Marine Science and Engineering, Qingdao Agricultural University, Qingdao 266109, China; 2 State Key Laboratory of Breeding Biotechnology and Sustainable Aquaculture, Institute of Oceanology, Chinese Academy of Sciences, Qingdao 266000, China; 3 Laboratory for Marine Biology and Biotechnology, Qingdao National Laboratory for Marine Science and Technology, Qingdao 266237, China; 4 Laboratory of Experimental Marine Biology, Institute of Oceanology, Chinese Academy of Sciences, Qingdao 266000, China; xjlubio@163.com

**Keywords:** hemocyte, innate immune memory, cell cycle, lipid metabolism, immune effector

## Abstract

Invertebrates rely exclusively on innate immunity but exhibit memory-like responses termed immune priming or trained immunity. In the commercially vital whiteleg shrimp (*Litopenaeus vannamei*), infection by *Vibrio parahaemolyticus* causes severe economic losses, yet the molecular networks driving secondary immune recall remain poorly understood. In this study, we established a two-step immune challenge model in *L. vannamei* using formaldehyde-inactivated *V. parahaemolyticus* and performed transcriptomic analysis on hemocytes to compare primary and secondary immune responses. Differentially expressed gene (DEG) screening and enrichment analyses (GO, KEGG, and GSEA) suggest that shrimp hemocytes undergo a broad and coordinated transcriptional reprogramming rather than uniform upregulation of immune genes. Transcriptomic data show potential associations between secondary immune priming and the modulation of cell fate processes: genes related to cell cycle progression (e.g., CDK1, CCNB3) and spindle assembly (e.g., MPS1) were significantly upregulated alongside apoptosis inhibition (CASP6 downregulation). Concurrently, metabolic remodeling was observed through the upregulation of lipid synthesis (SREBF1, FASN) and carbohydrate uptake pathways, potentially providing anabolic support for hemocyte growth and immune activation. Furthermore, the humoral effector responses appear to be strengthened, characterized by upregulated antimicrobial peptides (PEN, ALF) and the proPO melanization cascade (PPAF3, PPO3), whereas the expression of intracellular NLR was relatively suppressed, which might help mitigate excessive immune inflammation and immunopathological damage. Collectively, these transcriptomic findings identify a putative coordinated transcriptional signature of hemocyte recall responses in *L. vannamei*. This study expands our understanding of innate immune memory in invertebrates and provides candidate molecular markers for further study in disease-resistant breeding research in shrimp aquaculture.

## Simple Summary

Whiteleg shrimp are a key farmed seafood, but they frequently suffer severe disease and economic losses from *Vibrio parahaemolyticus* infection. Like other invertebrates, shrimp lack vertebrate-like adaptive immune memory, yet they can develop enhanced protection after an initial exposure, a response known as trained immunity. We investigated this defense by administering two sequential doses of formaldehyde-inactivated *Vibrio parahaemolyticus* and analyzing gene expression in shrimp hemocytes. We found that the second exposure triggered widespread gene expression changes: genes related to cell divide and grow, as well as immune effectors, were activated. The data may provide candidate genes for disease-resistant breeding research in shrimp aquaculture.

## 1. Introduction

Classical immunology has long restricted immune memory to the adaptive immune system of vertebrates, dismissing such capacity in the innate immune system of invertebrates and plants. This view has been increasingly challenged over recent decades [1,2,3,4]. Vertebrate adaptive immunity relies on somatic recombination of antigen receptors and persistent memory B and T lymphocytes to mediate highly specific immune recall [5]. In contrast, invertebrates and plants were historically considered to possess only non-specific, short-lived innate responses. Accumulating evidence now reveals that many invertebrates, including crustaceans, insects, molluscs, and cnidarians, exhibit immune priming, wherein prior exposure to pathogens or pathogen-derived molecules induces enhanced protection upon subsequent reinfection [2,6]. Similarly, vertebrates exhibit trained immunity, a form of innate immune memory characterized by long-term functional reprogramming of innate immune cells through epigenetic and metabolic modifications [7,8]. These findings highlight the evolutionary conservation of memory-like features within innate immunity across diverse taxa.

Crustaceans, as ecologically and economically significant invertebrates, rely solely on innate immunity to defend against pathogenic insults, making immune priming a particularly relevant phenomenon [9]. Early studies demonstrated that crayfish (*Parachaeraps bicarinatus*) primed with killed *Pseudomonas* bacteria showed reduced mortality upon secondary lethal challenge [10]. Subsequent research in copepods, shrimp, and crabs confirmed that crustacean immune priming can be strain-specific and involves both cellular and humoral pathways [11,12,13]. Studies demonstrated that shrimp administered heat-killed or formalin-inactivated *Vibrio alginolyticus* or *V. harveyi* exhibited enhanced immune parameters and higher survival upon secondary challenge with live pathogenic Vibrio [14,15]. Moreover, mixtures of inactivated Vibrio species can induce long-lasting immune protection for up to 8 weeks, accompanied by elevated phagocytic activity and disease resistance [14]. For commercially important species such as the whiteleg shrimp *Litopenaeus vannamei*, which suffers severe economic losses due to bacterial infections like vibriosis caused by *Vibrio parahaemolyticus* (VpAHPND) [14,16], elucidating the mechanisms underlying innate immune memory could provide crucial knowledge for developing novel disease control strategies and guiding selective breeding for pathogen resistance.

The phenomenology of invertebrate immune priming encompasses three distinct manifestations: sustained defense responses, rapid amplified recall responses, and shifts in immune mechanisms [2]. These responses are orchestrated by both conserved and lineage-specific mechanisms. Epigenetic modifications, including DNA methylation, histone acetylation/methylation, and non-coding RNA-mediated regulation, play central roles in encoding immune memory through stable modulation of gene expression without altering the underlying genome sequence [13,17]. For example, in the crab *Eriocheir sinensis*, increased glycolytic activity during secondary bacterial challenge is governed by KDM4-mediated H3K9me3 modifications at the promoters of metabolic genes [13]. In crustaceans and insects, the down syndrome cell adhesion molecule (Dscam) generates diverse isoforms via alternative splicing, functioning as an analog of vertebrate antibodies to mediate pathogen-specific recognition and immune priming [12,18,19]. Moreover, hemocytes, the primary immune cells in insects, undergo activation, differentiation, and transcriptional reprogramming during priming [15,20,21].

Most previous studies on secondary infection compared infected samples and uninfected controls at each time point, then contrasted differentially expressed genes (DEGs) from two infections. However, shrimp show substantial individual variation, and gene expression is affected by multiple factors. Uninfected samples at two different time points often show obvious differences in gene expression, which may lead to plenty of false-positive or false-negative results. In addition, most existing studies of secondary infection used live pathogens, whose proliferation after secondary challenge directly influences host gene expression, so the identified DEGs also include secondary responses caused by pathogen replication. To obtain a more comprehensive understanding of the transcriptional changes triggered by secondary infection, we used hemocytes of *L. vannamei* as the target tissue and employed formaldehyde-inactivated *V. parahaemolyticus* for both infections in this study. Hemocyte samples were collected at 24 h after the first immunization, and secondary immunization was performed at 7 days post-primary immunization, followed by hemocyte collection at 24 h after the second challenge. We directly performed comparative transcriptome sequencing between these two samples and analyzed their expression differences. Our objective was to identify key genes associated with innate immune memory that regulate the enhanced secondary response. The results will advance the molecular understanding of invertebrate-trained immunity and may contribute to practical applications in the selective breeding of disease-resistant shrimp.

## 2. Materials and Methods

### 2.1. Experimental Animals

Healthy whiteleg shrimp *L. vannamei* (body weight: 37.93 ± 3.33 g) were obtained from a commercial aquaculture farm and acclimatized in recirculating seawater systems (salinity 30‰, temperature 26 °C, dissolved oxygen > 5 mg L^−1^) for 7 days prior to experiments. Shrimp were fed a commercial pelleted diet twice daily and confirmed to be pathogen-free via bacterial culture of hemolymph before immunization.

### 2.2. Immune Challenge and Sample Collection

*Vibrio parahaemolyticus* (VpAHPND) was isolated from diseased shrimp, cultured in TSB + 2% NaCl medium at 28 °C with 200 rpm, then inactivated with 1% formaldehyde at 4 °C for 24 h; inactivation was verified by TCBS plate culture. Bacteria were washed three times in sterile PBS and adjusted to 4 × 10^8^ cfu/mL. The interval of 7 days between primary and secondary immunization was chosen based on previous studies showing that shrimp innate immune memory can be effectively induced within one week after priming with inactivated pathogens [15]. Hemocytes were sampled at 24 h post-secondary immunization to capture the peak transcriptional response of immune recall. A total of 60 individuals were injected with inactivated *V. parahaemolyticus* at a dose of 4 × 10^5^ cfu per shrimp. Hemocytes were collected 24 h after the first immunization (V1). Seven days after the first immunization, the remaining shrimp were injected with inactivated *V. parahaemolyticus* at 4 × 10^5^ cfu per shrimp for the second immunization, and hemocytes were collected 24 h after the second immunization (V2). Hemolymph was collected from shrimp ventral sinus using a sterile syringe preloaded with anticoagulant buffer (0.45 M NaCl, 0.1 M glucose, 30 mM trisodium citrate, 26 mM citric acid, 10 mM EDTA, pH 7.0) at a 1:1 ratio. Hemocytes were isolated by centrifugation at 800× *g* for 10 min at 4 °C, immediately frozen in liquid nitrogen, and stored at −80 °C for RNA extraction. Three biological replicates were prepared for each group, with 10 shrimp per replicate.

### 2.3. RNA Extraction and Transcriptome Sequencing

Total RNA was extracted from hemocyte samples using the TRIzol Reagent (Invitrogen, Carlsbad, CA, USA) following the manufacturer’s protocol. RNA purity, concentration, and integrity were assessed using a NanoDrop 2000 spectrophotometer (Thermo Fisher Scientific, Waltham, MA, USA), Qubit 3.0 Fluorometer (Invitrogen, Carlsbad, CA, USA), and Agilent 2100 Bioanalyzer (Agilent Technologies, Santa Clara, CA, USA), respectively. cDNA libraries were constructed using the NEBNext Ultra™ RNA Library Prep Kit for Illumina (NEB, Ipswich, MA, USA) and sequenced by Gene Denovo Biotechnology Co. (Guangzhou, China) using Illumina HiSeqTM 2500 (Illumina, San Diego, CA, USA). Raw sequencing data were filtered to remove low-quality reads (Q < 20), adapter sequences, and empty reads using Trimmomatic v0.39 to obtain clean reads, with an average of ~43 million clean reads generated per sample. Clean reads were then mapped to the *L. vannamei* reference genome (NCBI Assembly: GCF_042767895.1) using HISAT2 v2.2.1.

### 2.4. Differential Expressed Gene (DEG) Identification

Gene expression levels were quantified as Transcripts Per kilobase of exon model per Million mapped reads (TPM) using StringTie v2.1.7. DEGs between V1 and V2 groups were identified using the DESeq2 R package (v4.3.0) with the screening criteria: |log_2_(fold change, fc)| ≥ 1 and false discovery rate (FDR) < 0.05. Volcano plots were generated to visualize the global distribution of DEGs using the ggplot2 R package.

### 2.5. Functional Enrichment Analysis

Gene Ontology (GO) enrichment analysis of DEGs was performed using the ClusterProfiler R package (v4.6.0), with GO terms categorized into cellular component (CC), biological process (BP), and molecular function (MF). Significantly enriched GO terms were defined as those with FDR < 0.05, and the top 20 enriched terms were visualized in a dot plot. KEGG pathway enrichment analysis was conducted to annotate DEGs to biological pathways using the Kyoto Encyclopedia of Genes and Genomes (KEGG) database (https://www.genome.jp/kegg/, accessed on 21 August 2025) and ClusterProfiler. Significantly enriched KEGG pathways were identified with FDR < 0.05, and the top 10 pathways were plotted. Additionally, Gene Set Enrichment Analysis (GSEA) was performed to further validate the enrichment of functional gene sets in the KEGG database with the criterion of normalized enrichment score (NES) > 1 and FDR < 0.05.

### 2.6. Co-Regulatory Gene and Mechanistic Model Construction

Potential transcription factor (TF) target genes were predicted based on the *L. vannamei* transcription factor database and conserved TF binding sites in gene promoters (1.5 kb upstream of the transcription start site). Correlation analysis between TFs (PRDM1, E2F7, SREBF1) and their putative target genes was performed using the Pearson correlation coefficient (r > 0.8, *p* < 0.01). Based on DEG expression profiles, functional enrichment results, and TF-target gene correlations, a mechanistic model of the secondary immune response in shrimp hemocytes was constructed to illustrate the coordinated regulation of key biological processes.

### 2.7. Statistical Analysis

All experiments were performed with three biological replicates. Statistical analyses for gene expression and enrichment were conducted using R software (v4.3.1). Differences between groups were considered significant at *p* < 0.05, and multiple testing corrections were performed using the Benjamini–Hochberg method to calculate FDR.

## 3. Results

### 3.1. Global Identification of DEGs in Hemocytes Between Primary and Secondary Immunization

**Figure 1:**
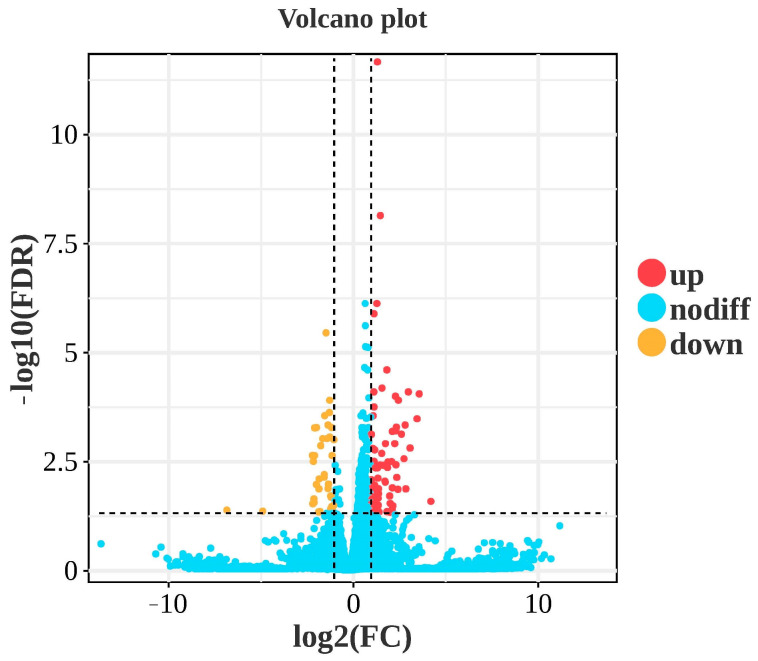
Screening of differential expressed genes (DEGs) from shrimp hemocytes between the primary and secondary immunization by *Vibrio parahaemolyticus*. Up and down represent up-regulated and down-regulated genes in shrimp hemocytes after secondary immunization by *Vibrio parahaemolyticus*, respectively. Nodiff represents genes with no differential expression between two groups.

Transcriptome sequencing of *L. vannamei* hemocytes from V1 (primary) and V2 (secondary) groups generated high-quality clean reads, with over 90% of reads mapped to the reference genome for each sample. Gene expression levels were normalized by TPM to eliminate the bias of gene length and sequencing depth, and a total of 116 DEGs were identified between the V1 and V2 groups (|log_2_fc| ≥ 1, FDR < 0.05), among which 78 genes were significantly upregulated, and 38 genes were significantly downregulated in the secondary immunization group (Figure 1). Global upregulation indicated a possible robust transcriptional reprogramming of hemocytes during secondary immune response to *V. parahaemolyticus*.

### 3.2. GO Enrichment Reveals Dominance of Cell Cycle and Mitotic Processes in DEGs

**Figure 2:**
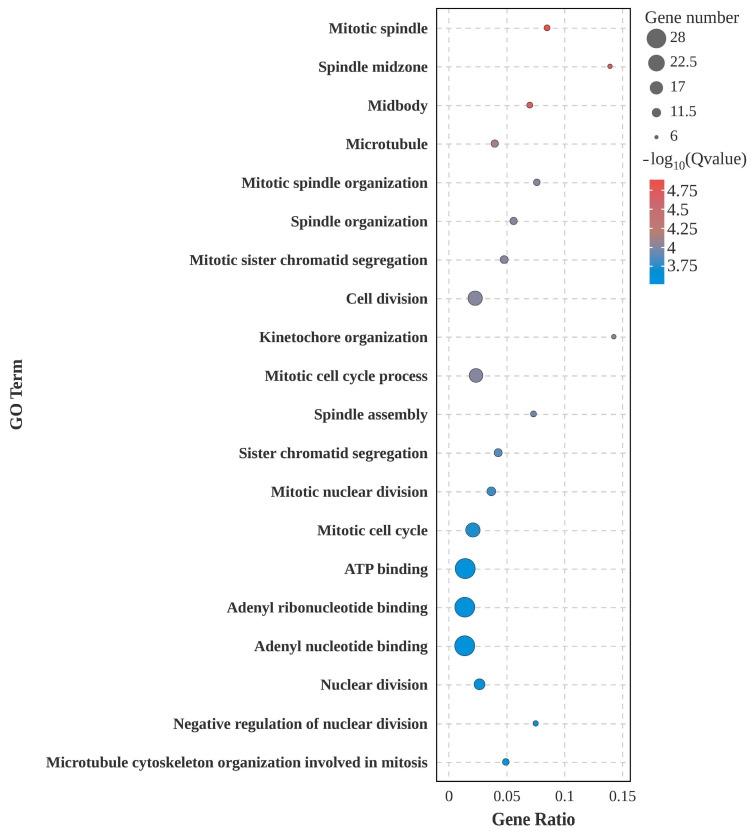
GO enrichment of DEGs from shrimp hemocytes between the primary and secondary immunization by *Vibrio parahaemolyticus*.

GO enrichment analysis of the 116 DEGs identified 20 significantly enriched terms (FDR < 0.05), with biological process (BP) as the most highly represented category, followed by cellular component (CC) (Figure 2). Mitotic and cell division-related terms were the most prominent in the BP category, including mitotic cell cycle process (GO:1903047), mitotic cell cycle (GO:0000278), cell division (GO:0051301), mitotic nuclear division (GO:0140014), sister chromatid segregation (GO:0000070), spindle organization (GO:0007051), and kinetochore organization (GO:0051383). For the CC category, core terms included microtubule (GO:0005874), mitotic spindle (GO:0072686), spindle midzone (GO:0051233), and midbody (GO:0030496), all of which are structural components critical for mitosis or cytokinesis. Molecular function (MF) enrichment was dominated by nucleotide binding terms (ATP binding, GO:0005524; adenyl ribonucleotide binding, GO:0032559), which may indicate the high energy demand of immune activation.

### 3.3. KEGG Enrichment Highlights Cell Cycle, Lipid Metabolism and Immune-Related Pathways

**Figure 3:**
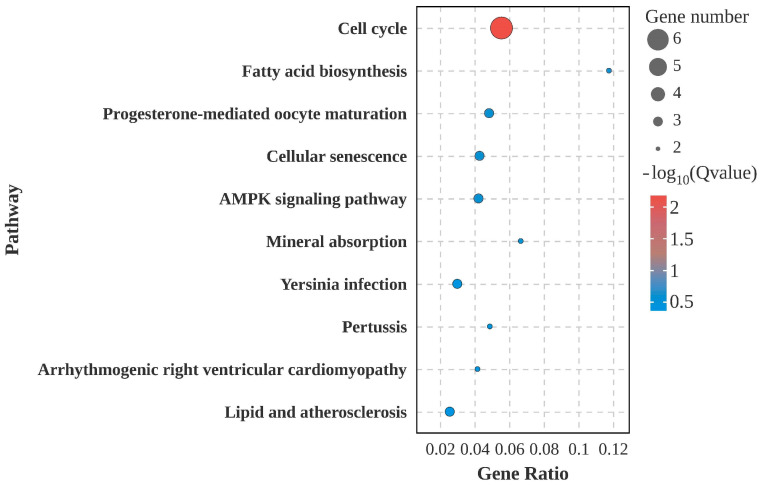
KEGG enrichment of DEGs from shrimp hemocytes between the primary and secondary immunization by *Vibrio parahaemolyticus*.

**Figure 4:**
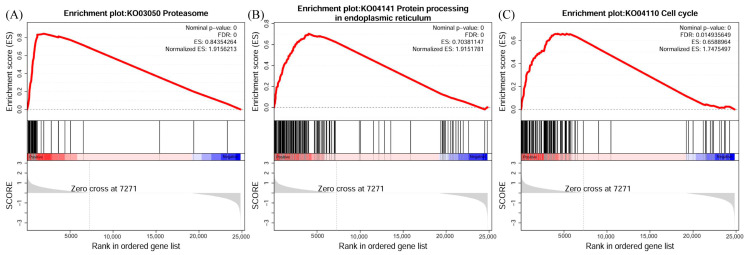
Significantly enriched KEGG pathways in shrimp hemocytes between the primary and secondary immunization determined by GSEA. Pathways including Proteasome (**A**), Protein processing in endoplasmic reticulum (**B**) and Cell cycle (**C**) were selected based on FDR < 0.05 and biological relevance to immunity.

KEGG enrichment analysis of DEGs identified 10 significantly enriched pathways (FDR < 0.05), spanning cellular processes, metabolism, and human diseases (Figure 3). The cell cycle pathway (ko04110) was the most significantly enriched, with the largest number of annotated DEGs, consistent with the GO enrichment results. Metabolic pathways were also prominently represented, including fatty acid biosynthesis (ko00061), which was the second most enriched pathway and the only core metabolic pathway in the top 10. Other enriched pathways included progesterone-mediated oocyte maturation (ko04914), cellular senescence (ko04218), and AMPK signaling pathway (ko04152)—all of which are associated with cell cycle regulation and metabolic control. Notably, pathogen infection-related pathways (Yersinia infection, ko05135) were also enriched, linking cellular/metabolic reprogramming to anti-pathogen immunity. GSEA further supported the significant enrichment of these core pathways (cell cycle, fatty acid biosynthesis), implying their potential functions in the secondary immune response (Figure 4).

### 3.4. DEGs Involved in Cell Cycle Progression and Cell Survival

A comprehensive set of cell cycle-related DEGs was identified and categorized into functional subclusters based on TPM-normalized expression levels, all of which exhibited significant upregulation (log_2_fc > 1, FDR < 0.05) except for two kinesin family genes (Table 1). Core cyclin and cyclin-dependent kinase genes (CCNB3, CCNA1, cdk1) and cell division cycle-associated gene (CDCA3) contribute to G2/M phase transition and cell cycle progression. MPS1 and Bub1b are key regulators of mitotic spindle checkpoint and chromosome segregation fidelity. A total of 17 DEGs were annotated to the function of kinetochore, spindle, and chromosome segregation, such as ASPM, CENP-F, Klp61F, Cenpe, NDC80, Nuf2, etc. Genes governing cleavage furrow formation and cytokinesis (IQGAP3, Arhgap19, CIT, PRC1) will facilitate complete cell division. Histone genes (H1, H2A) and condensin subunit (NCAPD2) support DNA replication and chromatin stability. Transcription factors (E2F7, PRDM1) and splicing regulator (SRRM2) are related to cell fate and differentiation, suggesting hemocyte phenotypic differentiation during secondary immunization. The most highly upregulated gene in this category was TACC2 (log_2_fc = 3.49), a key regulator of cell proliferation and cell survival; additional pro-survival genes (PBK, IGFBP-1/2, HSC70-3) were also upregulated. The pro-apoptotic gene Casp6 (log_2_fc = −1.33) was the only significantly downregulated gene, suggesting that the apoptosis process may be inhibited.

### 3.5. DEGs Orchestrating Lipid Metabolism Reprogramming

Lipid metabolism-related DEGs were exclusively upregulated in hemocytes during the secondary immunization and categorized into fatty-acid synthesis and substrate supply (Table 2), consistent with the KEGG enrichment of fatty acid biosynthesis. Fatty-acid synthesis-related genes include the master transcription factor of lipogenesis, SREBF1 and the rate-limiting enzyme of de novo fatty acid synthesis, Fasn. In addition, ACSBG2 functions to activate fatty acids to acyl-CoA for membrane biogenesis. Substrate supply-related genes include carbohydrate transport and metabolism genes (SGL, SLC23A1, SLC5A1), potentially providing carbon skeletons and energy for de novo lipid synthesis and cell growth.

### 3.6. DEGs Regulating the Humoral Immune Effector Response

Immunity-related DEGs were categorized into the proPO system, immune effectors, and immune activation clusters (Table 3). All core components of the proPO cascade (PPAF1/2/3, PPO3) were upregulated, enhancing the melanization response for pathogen encapsulation and killing. Key antimicrobial peptide (AMP) genes (PEN, ALF, Arasin, Cru) were significantly upregulated, strengthening soluble antimicrobial defense against *V. parahaemolyticus*. Extracellular pathogen recognition and processing genes (FCN1, ENPP3) were upregulated, while the intracellular receptor NLR was downregulated.

### 3.7. Core Transcription Factors and the Proliferation-Metabolism-Effector Synergistic Model

**Figure 5:**
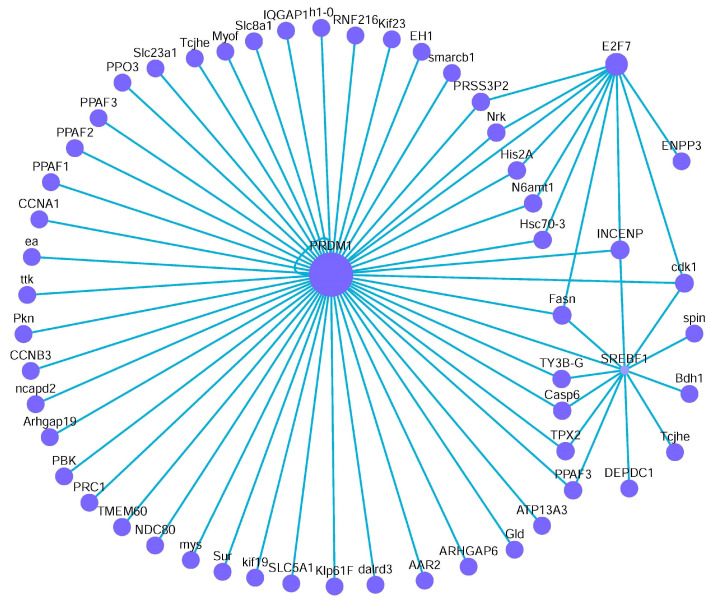
Identification and correlation analysis of potential target genes for transcription factors PRDM1, E2F7 and SREBF1. TFs (PRDM1, E2F7, SREBF1) were selected by correlation analysis (r > 0.8, *p* < 0.01) and functional relevance; the network illustrates their putative regulatory roles linking proliferation, metabolism, and immunity.

Based on gene expression data, transcription factor target gene prediction and correlation analysis, we identified PRDM1, E2F7, and SREBF1 as core regulatory TFs for the secondary immune response (Figure 5). E2F7 regulates the expression of cell cycle genes (CDK1, CCNA1, CCNB3), SREBF1 controls lipid metabolism genes (Fasn, ACSBG2), and PRDM1 modulates both cell fate genes and immune effector genes, forming a coordinated TF regulatory network that links cell proliferation, metabolism, and immunity.

## 4. Discussion

Our transcriptomic data suggest that secondary *Vibrio parahaemolyticus* challenge induces broad hemocyte reprogramming, rather than uniform immune gene upregulation. At a system level, the observed changes are broadly consistent with a coordinated secondary-response state involving increased cell fate control, metabolic remodeling that may support biosynthesis, and strengthened humoral effector potential.

A substantial fraction of upregulated genes is linked to cell-cycle progression, including CDK1, CDCA3, CCNA1, and CCNB3, as well as spindle-checkpoint regulators such as MPS1 and BUB1B. Genes involved in kinetochore function, spindle assembly, and chromosome segregation (NDC80, NUF2, CENPE, CENPF, INCENP, ESPL1, DEPDC1, TPX2, NUMA1, ASPM, and kinesins) were also elevated. Although these genes are related to cell-cycle activity, some of them play important roles in cell survival [22,23,24,25]. Circulating *L. vannamei* hemocytes are generally non-proliferative under physiological conditions. During secondary infection, the increase in shrimp circulating hemocytes is mainly from hematopoietic tissues [26]. However, differentiation of immune cell populations has been implicated in mosquito immune memory-like responses [27]. Upregulation of cytokinesis-related genes (PRC1, CIT, ARHGAP19, IQGAP3) might also contribute to hemocyte homeostasis [28]. Meanwhile, increased expression of genome-maintenance-associated genes (NCAPD2, RNASEH2B, and histone-related genes) may imply adaptive responses to inflammatory stress [29,30]. We also observed altered expression of genes linked to fate regulation (E2F7/8, PRDM1, SRRM2), suggesting that secondary challenge may involve qualitative state transitions in hemocytes, in addition to potential changes in abundance [31,32]. The concurrent upregulation of several survival-associated genes (PBK, IGFBP1/2, TACC2, CHF, Hsc70-3, SPON2, KCP) and downregulation of CASP6 further support a potential shift toward survival-maintenance states [33,34]. Collectively, the upregulation of some genes might be associated with maintaining hemocyte viability, whereas the biological roles of other genes related to cell fate remain unclear in shrimp secondary immune response. Future RNAi experiments will be carried out to verify their regulatory roles in shrimp innate immune memory.

Metabolic signatures in the secondary response further suggest a coordinated anabolic-support program. Upregulation of SREBF1 and FASN, together with KEGG enrichment of fatty acid biosynthesis, suggests a possible enhancement of *de novo* lipogenesis, which in other systems supports membrane production and cell growth [35]. Increased ACSBG2 expression may suggest strengthened fatty-acid activation to acyl-CoA, potentially supporting membrane assembly, storage, and energy-linked pathways [36]. We also detected increased expression of carbohydrate transport/metabolism-related genes (SLCs, sgl/UGDH), which may reflect increased substrate supply to sustain biosynthetic flux [37,38]. Collectively, these transcript-level changes are compatible with a model of coupled lipid synthesis and utilization that may support hemocyte survival during secondary challenge. This viewpoint and noted dedicated metabolic assays are needed for further verification.

Immune-effector transcriptional patterns indicate a tendency toward enhanced humoral defense in the secondary response. Multiple components of the prophenoloxidase (proPO) cascade (PPAF1, PPAF2, PPAF3, PPO3) were upregulated, suggesting increased melanization-related activation potential [39,40,41]. We also observed increased expression of antimicrobial peptide genes (PEN, ALF, Arasin, Cru), consistent with strengthened soluble antimicrobial capacity [42,43,44,45]. Upregulation of Fcn1-like lectin and ENPP3-like phosphodiesterase may imply reinforced extracellular recognition/processing components [46,47]. In contrast, NLR was downregulated, suggesting that recall responses may involve selective pathway tuning rather than global activation of all immune branches [48,49]. In the present study, two sequential inactivated bacterial stimulation rather than persistent continuous antigen exposure, typical induction of immune tolerance, was used to prepare experimental samples. Therefore, we tend to attribute NLR downregulation to active fine-tuning of trained immunity, instead of passive immune tolerance. One possible interpretation is that the host restrains excessive intracellular NLR-mediated inflammatory signaling while amplifying extracellular humoral immune cascades to avoid immunopathological damage during recall response. Further NLR functional studies will distinguish between immune tolerance and trained immunity regulation in future research.

Some weakly enriched or human disease-related KEGG pathways were observed in the analysis. Given the evolutionary divergence between shrimp and humans, these pathways were interpreted cautiously, and only those with clear homology to cell fate, metabolism, or immune processes were considered relevant to the secondary immune response in shrimp. Some other limitations should also be noticed. Formaldehyde fixation indeed changes partial surface protein conformation and modifies partial PAMPs, which may induce non-natural immune pathways different from heat-inactivated or live pathogens, which possess restricted direct application value for natural vibriosis prevention in practical shrimp farming. The differential genes identified between V1 and V2 cannot completely exclude partial residual long-lasting primary immune effects. The time-matched single-challenge control group (sampled on day 8 post single injection) is recommended to filter true trained immunity recall genes. In addition, without a complete dose–response test, we cannot fully eliminate the risk that the selected dose triggers partial immune exhaustion rather than specific trained immunity priming, which may interfere with transcriptomic profiling results. Finally, the obtained transcriptional differences may originate from two aspects: intrinsic transcriptional reprogramming inside individual hemocytes or dynamic shifts in the proportional composition of different hemocyte subsets. Follow-up single-cell transcriptome sequencing could be used to separate these two regulatory modes. Although several issues remain to be further clarified in the present study, our findings provide new insights into the regulatory mechanisms of innate immune memory in invertebrates.

## 5. Conclusions

Overall, our data suggest a tentative “cell survival–metabolism–effector” model for secondary hemocyte responses in *L. vannamei*. In this model, cell survival modules, metabolic-support pathways, and humoral effectors are co-regulated during re-challenge. These conclusions reflect transcriptomic associations, and future work combining hemocyte profiling, functional perturbation, and infection-based validation will clarify their roles in innate immune memory-like protection.

## Figures and Tables

**Table 1:** Expression profile of DEGs involved in cell fate.

Functional Categories	Gene ID	Symbol	Description	log2FC	FDR
Cell cycle	ncbi_113829902	CCNB3	G2/mitotic-specific cyclin-B3	3.11	0.0016
	ncbi_113821735	CCNA1	G2/mitotic-specific cyclin-A	2.04	0.0300
	ncbi_113818305	cdk1	cyclin-dependent kinase 1	1.77	0.0098
	ncbi_113829899	CDCA3	cell division cycle-associated protein 3	1.01	0.0317
	ncbi_113804403	MPS1	monopolar spindle 1	2.45	0.0142
	ncbi_113820973	Bub1b	mitotic checkpoint serine/threonine-protein kinase BUB1	2.28	0.0013
	ncbi_113819387	ASPM	abnormal spindle-like microcephaly-associated protein homolog	2.78	0.0028
	ncbi_113810390	CENP-F	centromere protein F	2.65	0.0008
	ncbi_113803105	Klp61F	kinesin-like protein at 61F	2.37	0.0005
	ncbi_138865048	ASPM	abnormal spindle-like microcephaly-associated protein homolog	2.34	0.0006
	ncbi_113813509	Cenpe	centromere-associated protein E	2.32	0.0001
	ncbi_113806815	ESPL1	putative separin	2.18	0.0341
	ncbi_113805080	DEPDC1	DEP domain-containing protein 1A	1.86	0.0000
	ncbi_113827798	INCENP	inner centromere protein A	1.75	0.0092
	ncbi_113823285	NUMA1	nuclear mitotic apparatus protein 1-like	1.66	0.0040
	ncbi_113824558	Nuf2	kinetochore protein Nuf2	1.42	0.0470
	ncbi_113823574	NDC80	kinetochore protein NDC80 homolog	1.38	0.0331
	ncbi_113825603	TPX2	targeting protein for Xklp2 homolog	1.38	0.0227
	ncbi_113809463	Kif19	kinesin family member 19A	−1.73	0.0014
	ncbi_113821854	Kif23	kinesin family member pavarotti	3.02	0.0001
	ncbi_113809404	E2F7	transcription factor E2F8	1.86	0.0461
	ncbi_113820103	PRDM1	PR domain zinc finger protein 1	1.23	0.0427
	ncbi_113830047	SRRM2	serine/arginine repetitive matrix protein 2-like	1.01	0.0207
Cytokinesis	ncbi_113817373	IQGAP3	ras GTPase-activating-like protein IQGAP3	2.84	0.0005
	ncbi_113817406	Arhgap19	Rho GTPase activating protein at 54D	2.39	0.0076
	ncbi_113808119	CIT	citron rho-interacting kinase-like	2.11	0.0033
	ncbi_113805259	PRC1	protein regulator of cytokinesis 1	1.39	0.0179
Genome maintenance and replication-stress response	ncbi_113821142	H1	histone H1-beta	1.59	0.0001
	ncbi_113823508	NCAPD2	CAP-D2 condensin subunit	1.11	0.0003
	ncbi_113829109	H2A	histone H2A-like	1.10	0.0017
	ncbi_113823352	RNaseH2B	ribonuclease H2 subunit B	−1.24	0.0001
Cell survival	ncbi_113829453	TACC2	transforming acidic coiled-coil-containing protein 2	3.49	0.0003
	ncbi_113826652	PBK	lymphokine-activated killer T-cell-originated protein kinase	2.02	0.0474
	ncbi_113808590	IGFBP-2	single insulin-like growth factor-binding domain protein-2	2.01	0.0201
	ncbi_113808589	CHF	crustacean hematopoietic factor-like protein	1.39	0.0138
	ncbi_113815329	Spon2	M-spondin	1.36	0.0429
	ncbi_138860105	KCP	kielin/chordin-like protein	1.18	0.0415
	ncbi_113818927	HSC70-3	heat shock protein 70 cognate 3	1.16	0.0002
	ncbi_113813598	IGFBP-1	insulin-like growth factor-binding protein-related protein 1	1.15	0.0000
Apoptosis	ncbi_113817804	Casp6	caspase-6 isoform X1	−1.33	0.0005

**Table 2:** Expression profile of DEGs involved in lipid metabolism.

Functional Categories	Gene ID	Symbol	Description	log2FC	FDR
fatty-acid synthesis	ncbi_113829801	SREBF1	Sterol regulatory element binding protein	1.34	0.0000
	ncbi_113815940	Fasn	fatty acid synthase	3.60	0.0001
	ncbi_113800965	ACSBG2	acyl-CoA synthetase bubblegum family member 1	1.50	0.0000
substrate supply	ncbi_113817433	SGL	UDP-glucose 6-dehydrogenase sgl	1.02	0.0259
	ncbi_113800810	SLC23A1	solute carrier family 23 member 1	1.78	0.0013
	ncbi_113828571	SLC5A1	sodium/mannose cotransporter SLC5A10	1.15	0.0415

**Table 3:** Expression profile of DEGs related to immunity.

Functional Categories	Gene ID	Symbol	Description	log2FC	FDR
proPO system	ncbi_113822282	PPAF3	venom protease	1.37	0.0079
	ncbi_113828683	PPAF1	venom protease	1.31	0.0346
	ncbi_113827090	PPO3	phenoloxidase 3	1.27	0.0136
	ncbi_113802490	PPAF2	phenoloxidase-activating factor 2	1.08	0.0265
	ncbi_113826214	PPAF3	CLIP domain-containing serine protease HP8	1.05	0.0415
Immune effectors	ncbi_113808997	PEN	penaeidin-3b-like	1.34	0.0046
	ncbi_113800363	ALF	anti-lipopolysaccharide factor	1.31	0.0000
	MSTRG.10452	Arasin	arasin-like protein	1.16	0.0032
	ncbi_113820206	Cru	type III crustin cruIII-3	1.12	0.0172
Immune activation	ncbi_138860764	FCN1	ryncolin-1-like	1.04	0.0136
	ncbi_113806994	ENPP3	venom phosphodiesterase	2.15	0.0132
	ncbi_113812252	NLR	NLR family CARD domain-containing protein 4-like	−1.14	0.0005

## Data Availability

The original contributions presented in the study are included in the article. Further inquiries can be directed to the corresponding author.
